# Editorial: Biomaterials and biological regulation for bone tissue remodeling and regeneration

**DOI:** 10.3389/fbioe.2025.1582215

**Published:** 2025-04-02

**Authors:** Xianrui Yang, Masashi Nagao, Chen-He Zhou

**Affiliations:** ^1^ Department of Orthodontics, University of Florida College of Dentistry, Gainesville, FL, United States; ^2^ Department of Orthopaedics, Juntendo University School of Medicine, Tokyo, Japan; ^3^ Medical Technology Innovation Center, Juntendo University, Tokyo, Japan; ^4^ Department of Orthopedics Surgery, The Second Affiliated Hospital, Zhejiang University School of Medicine, Hangzhou, China

**Keywords:** biomaterials, biological regulation, bone regeneration, bone remodeling, signaling/signaling pathways

Bone tissue consists of an organic matrix, primarily collagen type I, and an inorganic mineral phase, mainly hydroxyapatite, which together provide bone strength, flexibility, and function (Šromová et al., 2023). Numerous cells are involved in the maintenance and dynamics of bone tissue. For instance, osteocytes embedded in the bone matrix function as mechanosensors and regulators, coordinating the activity of other cells in response to mechanical and biochemical signals (Delgado-Calle and Bellido, 2022). Mesenchymal stem cells (MSCs), located in the bone marrow and periosteum, are multipotent cells that can differentiate into osteoblasts and chondrocytes (Sheng, 2015). In addition to MSCs, osteogenic precursor cells within the periosteum also play a significant role in bone formation (Donsante et al., 2021). Furthermore, skeletal stem cells (SSCs) have been identified as a distinct population within bone tissue that possesses the ability to self-renew and generate various cell types, including osteoblasts, chondrocytes, and stromal cells (Yuan et al., 2022).

The bone remodeling process is continually active throughout life by the coordinated activities of osteoclasts and osteoblasts, which are essential for maintaining bone homeostasis and adapting to mechanical stresses (Bolamperti et al., 2022). For instance, during orthodontic tooth movement, alveolar bone remodeling occurs due to the actions of osteoblasts on the tension side and osteoclasts on the compression side. The process by which cells respond to mechanical forces is known as mechanotransduction, induced by mechanosensors such as those on osteocytes that detect mechanical loads and translate them into biochemical signals (Li et al., 2021). The bone regeneration process is reparative and occurs in response to injury and involves inflammation, soft callus formation, hard callus formation, and bone formation (Duda et al., 2023). The initial inflammatory phase activates immune cells, such as macrophages, and releases cytokines to recruit MSCs to the injury site (Duda et al., 2023). The subsequent phases involve the differentiation of MSCs into osteoblasts and chondrocytes to form new bone and synthesize a new bone matrix (Donsante et al., 2021). The regulation of biological signaling and the use of biomaterials play crucial roles in these processes.

Some essential biological mediators, including morphogenetic proteins (BMPs), transforming growth factor-beta (TGF-β), insulin-like growth factors (IGFs), vascular endothelial growth factors (VEGFs), receptor activator of nuclear factor kappa-B ligand (RANKL), and osteoprotegerin (OPG), are extensively studied in the process of bone turnover (Bartold and Ivanovski, 2025). Osteoblasts and stromal cells produce RANKL, which binds to the RANK receptor on osteoclast precursors, promoting their differentiation into mature osteoclasts (Udagawa et al., 2021). The decoy receptor OPG, secreted by osteoblasts, competes with RANKL to prevent excessive osteoclast activation, thereby maintaining bone dynamics (Udagawa et al., 2021). Additionally, systemic regulators like parathyroid hormone (PTH) and vitamin D influence calcium homeostasis and bone metabolism, stimulating osteoblast activity through RANKL (Russell, 2024). PTH has a dual role in these processes: It promotes bone formation with intermittent administration while leading to bone resorption with chronic elevation (Liu et al., 2024). Furthermore, inflammatory cytokines such as tumor necrosis factor-alpha (TNF-α) and interleukins IL-6 and IL-1β play dual roles as well, initially promoting inflammation and bone resorption but later facilitating repair by recruiting progenitor cells (Yao et al., 2024).

Mechanosensors on the cell surface can convert mechanical stimuli into biochemical signals to regulate cellular responses to induce bone remodeling. For instance, piezo channels, especially piezo1 and piezo2, are found in osteoblasts, osteocytes, and mesenchymal stem cells (Xu et al., 2021). The activation of piezo1 by mechanical stress causes calcium influx, triggering downstream signaling pathways such as Wnt/β-catenin and Yes-associated protein/transcriptional coactivator with PDZ-binding motif (YAP/TAZ), along with the release of signaling molecules like prostaglandins and nitric oxide, which influence the activity of osteoblasts and osteoclasts (Huang et al., 2023). Research also indicates that in osteocytes, piezo channels regulate mechanotransduction by modulating sclerostin expression, thus affecting bone resorption through osteoclast regulation (Huang et al., 2023). Furthermore, piezo1-mediated signaling affects MSC differentiation toward osteogenic lineages while inhibiting adipogenesis to enhance bone regeneration (Huang et al., 2023).

Various biomaterials have been developed and applied to enhance bone formation. Bioactive ceramics, such as hydroxyapatite (HA) and tricalcium phosphate (TCP), mimic the mineral composition of bone, improving osteointegration and stability (Juhasz and Best, 2012). Polymeric biomaterials, including natural types like collagen, chitosan, and alginate, as well as synthetic types like polycaprolactone (PCL), polylactic acid (PLA), and polyglycolic acid (PGA), provide tunable mechanical properties, biocompatibility, and degradation rates, making them suitable for a range of orthopedic applications (Asti and Gioglio, 2014). Composite biomaterials combine ceramics and polymers, further optimizing bioactivity and mechanical integrity (Vahidi et al., 2024). Moreover, scaffolds can be functionalized with growth factors, drugs, or peptides to enhance their regenerative capabilities. For instance, BMP2-loaded scaffolds have been widely utilized in clinical settings to promote bone formation in critical-sized bone defects (Chen et al., 2021).

Emerging technologies such as 3D bioprinting and gene editing are increasingly being utilized to create patient-specific solutions, allowing precise control over scaffold architecture and bioactive properties for bone regeneration (Lee et al., 2024). Nanomaterials, including nanohydroxyapatite and graphene-based substances, provide superior bioactivity and mechanical strength by mimicking the nanoscale structure of the bone matrix while also serving as carriers for growth factors, genes, and drugs (Chinnaiyan et al., 2024). Additionally, bioactive coatings that incorporate antimicrobial agents, peptides, or stem cell-derived exosomes further enhance the regenerative potential of biomaterial implants (Agnihotri et al., 2024). Furthermore, DNA hydrogels represent innovative emerging biomaterials and show significant promise as bone-promoting scaffolds, as demonstrated by researchers in mouse calvarial regeneration (Athanasiadou et al., 2023).

Although extensive research has been conducted to improve bone regeneration and remodeling, challenges persist in translating laboratory findings into clinical therapies for patients. To ensure successful outcomes, several factors, including biomaterial degradation rates, immune compatibility, and cost-effectiveness, need to be addressed. Moreover, the field is shifting towards personalized medicine approaches, where patient-specific factors inform the selection of biomaterials, stem cells, and therapeutic strategies. Advanced bioprinting and tissue engineering techniques have the potential to create custom scaffolds with precise architectural and biological properties. With the advancement of microfluidic devices, organ-on-a-chip models have also been utilized in the bone field, such as a bone-on-a-chip platform that simulates the dynamic biological processes of bone remodeling and mineralization (Mansoorifar et al., 2021), which could provide a personalized testing platform for treating patients with bone diseases.

The six papers collected for our special issues are all discussing about novel biomaterials for bone tissue regeneration (Kitahara et al.; Lei et al.; Wang et al.; El-Nablaway et al.; El-Nablaway et al.; Indurkar et al.). Lei et al. built rabbit critical-size bone defects and tested porous calcium-phosphate (CaP) ceramics for bone regeneration, and they found that CaP ceramics can improve bone-forming ability with adequate time (Lei et al.). Wang et al. established SD-rat calvarial critical-sized defects and tested the effect of α-calcium sulfate hemihydrate/treated detin matrix composite, and they suggest that the composite cement has promising potential to be an alternative for bone regeneration (Wang et al.). Kitahara et al. created a novel rat femoral nonunion model and tested rhBMP-2-loaded hydroxyapatite/betatricalcium phosphate microsphere/hydrogel composite and indicated it significantly improved bone union rates and new bone formation (Kitahara et al.). El-Nablaway et al. reviewed the locally applied repurposed pharmaceuticals for periodontal tissue regeneration with their success and drawbacks to help explore the effectiveness and efficiency, economical, and state topical pharmaceutical preparations (El-Nablaway et al.). They also reviewed the cutting-edge bioactive polymeric hydrogels for periodontal regeneration to help establish prospective clinical applications (El-Nablaway et al.). Indurkar et al. use gelatin methacrylate and citrate-containing amorphous calcium phosphate to develop nanocomposite hydrogel (Indurkar et al.). All these studies provide novel information in the field of bone regeneration and help advance its application for clinical treatment in the future.
